# Computational modeling of the p7 monomer from HCV and its interaction with small molecule drugs

**DOI:** 10.1186/2193-1801-2-324

**Published:** 2013-07-18

**Authors:** Yi-Ting Wang, Hao-Jen Hsu, Wolfgang B Fischer

**Affiliations:** Institute of Biophotonics, School of Biomedical Science and Engineering, National Yang-Ming University and Biophotonics & Molecular Imaging Research Center (BMIRC), National Yang-Ming University, Taipei, 112 Taiwan; Department of Life Science, Tzu Chi University, Hualien, 970 Taiwan

**Keywords:** p7 protein, HCV, Membrane protein, Ion channels, Molecular dynamics simulations, Docking approach

## Abstract

Hepatitis C virus p7 protein is a 63 amino acid polytopic protein with two transmembrane domains (TMDs) and one of the prime targets for anti HCV drug development. A bio-inspired modeling pathway is used to generate plausible computational models of the two TMDs forming the monomeric protein model. A flexible region between Leu-13 and Gly-15 is identified for TMD1_1-32_ and a region around Gly-46 to Trp-48 for TMD2_36-58_. Mutations of the tyrosine residues in TMD2_36-58_ into phenylalanine and serine are simulated to identify their role in shaping TMD2. Lowest energy structures of the two TMDs connected with the loop residues are used for a posing study in which small molecule drugs BIT225, amantadine, rimantadine and *N*N-DNJ, are identified to bind to the loop region. BIT225 is identified to interact with the backbone of the functionally important residues Arg-35 and Trp-36.

**Electronic supplementary material:**

The online version of this article (doi:10.1186/2193-1801-2-324) contains supplementary material, which is available to authorized users.

## Background

Computational methods have grown to a stage where they can be used to build small proteins or at least certain parts of larger proteins, with respectably good results. Software has been developed which allows small sized proteins to be ‘built’ with high resolution (Rohl et al. 2004a; Rohl et al. 2004b; Kim et al. 2004; Kaufmann et al. 2010). Building assemblies of small membrane proteins, approaches have been adopted which include a combination of molecular dynamics simulations and docking protocols in various ways (Bowie 1997; Kukol & Arkin 1999; Kerr et al. 1996; Forrest et al. 2000; Cordes et al. 2001; Bowie 2005; Patargias et al. 2006; Psachoulia et al. 2008; Krüger & Fischer 2009; Park et al. 2012). A major obstacle, is to assemble proteins with oligomeric TMD topology. Simplified, but nevertheless bio-inspired routes have to be defined while assembling the proteins using a computational approach (Hsu & Fischer 2011).

The routes of assembly are guided by the knowledge of how membrane proteins are inserted or ‘folded’ into the lipid membrane. Membrane proteins are translated at the endoplasmic reticulum with the help of translocons (Johnson & van Waes 1999; Rapoport et al. 2004; Cheng & Gilmore 2006). The translocons are membrane spanning proteins which enable the primary sequence of a membrane protein to be folded into the secondary structure within the hydrophobic environment of the lipid membrane. The topology of the respective membrane protein is generated according to the information encoded in its primary sequence (von Heijne 1988; Hessa et al. 2005; Fink et al. 2012). The protein is then finally released into the lipid bilayer. The ‘monomeric unit’ is the protein, which needs to be assembled further into the quaternary fold. Within the lipid membrane, the fold of a helical motif is adopted by the membrane protein prior to any consecutive steps like assembly or integration of co-factors (Popot & Engelman 1990; Engelman et al. 2003). Therefore, once the secondary structure is formed, the protein remains in this fold.

Viral channel forming proteins (Fischer & Sansom 2002; Gonzales & Carrasco 2003; Fischer & Krüger 2009; Nieva et al. 2012) are candidate proteins which can be built along these considerations using computational techniques (Krüger & Fischer 2009; Hsu & Fischer 2011). Viral channel forming proteins are found as bitopic and polytopic membrane proteins with up to three TMDs (Hsu & Fischer 2011; Fischer & Krüger 2009; Wang et al. 2010). What they all have in common, is their existence as homo-oligomers with a minimum number of four monomeric units in order to be totally functional. Their biophysical role is identified as to alter chemical or substrate gradients across the lipid bilayer. However, the function within the infectivity cycle of the individual viruses still remains mostly to be discovered.

Being one of the viral channel forming proteins, encoded by HCV, p7 is built along the outlined computational road. The genome of HCV is expressed as a large polyprotein which is cleaved by proteases into the array of individual proteins posttranslational. The polytopic p7 protein is located at the border between the structural protein E2 and the following non-structural proteins (Lin et al. 1994). With its 63 amino acids, it has been suggested to have two TMDs (Patargias et al. 2006; Carrère-Kremer et al. 2002). Channel activity of the protein has been reported (Pavlovic et al. 2003; Premkumar et al. 2004; Chew et al. 2009; Griffin et al.; 2003; Clarke et al. 2006), as well as the effect of potential drugs on its channel activity (Pavlovic et al. 2003; Premkumar et al. 2004; Griffin et al. 2003; Steinmann et al. 2007a). The role of the protein within the infectivity cycle is proposed to be similar to M2 of influenza A in alternating the pH gradient across lipid membranes (Griffin 2009).

A helical TM motif is confirmed by NMR spectroscopy for a peptide corresponding to the second TMD (Cook & Opella 2010) and a hair-pin structure for a full length protein (Cook & Opella 2011). Detailed NMR experiments identify TMD1 consisting of two helical parts including the first 15 residues (Cook & Opella 2011; Montserret et al. 2010), as well as TMD2 (Cook & Opella 2011). The oligomeric state of p7 is suggested to be hexameric based on electron microscopic data (Griffin et al. 2003; Luik et al. 2009), with a potential to form heptameric assemblies as well (Clarke et al. 2006). It is most likely, that there is a strong strain specific aspect to assembly and drug sensitivity (StGelais et al. 2009). In a NMR spectroscopic study an all atom hexameric bundle structure is reported for the first time (OuYang et al. 2013). Computational methods have been done to generate a hexamer (Patargias et al. 2006). Conductance studies with liposome based essays of a set of mutant p7 reveal a concerted action of all structural elements (StGelais et al. 2007). The TMDs and the basic loop are important for the proper functioning of the channel.

It is assumed that the individual TMDs envision a short period of conformational equilibration within the lipid environment prior to assembling into the oligomer. Along this bio-inspired pathway, structural integrity of the individual TMDs of p7 is evaluated using molecular dynamic (MD) simulations in a fully hydrated lipid bilayer. The sequence of the TMDs used, is based on a bioinformatics approach (Patargias et al. 2006). A series of mutations have been done in TMD2 to evaluate the role of Tyr-42 in the fold of helix.

## Materials and methods

The sequence of the TMDs of p7 was taken from the HCV genotype 1a, H77 strain (Pavlovic et al. 2003): ALENLVILNA^10^ ASLAGTHGLV^20^ SFLVFFCFAW^30^ YLKGRWVPGA^40^ VYAFYGMWPL^50^ LLLLALPQR^60^ AYA. The following systems have been used in this study: TMD1_10-32_, TMD1_1-32_, and TMD2_36-58_, M_L_ (monomer p7 with loop, residues 10–57). The assembled monomer, TMD1 and TMD2 without a loop (no loop), is named ‘M_NL_’.

The following mutations in TMD2 were generated: TMD2_36-58_Y42/45F, TMD2_36-58_Y42/45S, and TMD2_36-58_F44Y.

The transmembrane domains TMD1 and TMD2 were generated as ideal helices using the MOE software package (Molecular operating environment, http://www.chemcomp.com).

### MD simulations

Lipid bilayer patches were generated from 16:1–18:1 Diester PC, 1-Palmitoyl-2-Oleoyl-sn-Glycero-3-Phosphocholine (POPC) molecules on the basis of the parameters of (Chandrasekhar et al. 2003) as reported earlier (Krüger & Fischer 2008). The lipid system, which included 128 lipid and 3655 water molecules was due to a 70-ns MD simulation. For simulations of the p7 monomer, four of these lipid patches were combined to generate a larger patch of 288 lipid molecules and 8748 water molecules. The larger patch was equilibrated for 50 ns.

MD simulation of the systems, reported in the present study, were carried out with GROMACS 4.0.7, using Gromos96 (ffG45a3) force field. The temperature of the peptide, lipid, and the water molecules were separately coupled to a Berendsen thermostat at 310K with a coupling time of 0.1 ps. For simulating the individual TMDs, a fully isotropic pressure coupling was applied with a coupling time of 1.0 ps and a compressibility 4.5e-5 bar^-1^. The monomer was simulated with a semi isotropic pressure coupling scheme. Long range electrostatics had been calculated using the particle-mesh Ewald (PME) algorithm with grid dimensions of 0.12 nm and interpolation order 4. Lennard-Jones and short-range Coulomb interactions were cut off at 1.4 and 1nm, respectively.

Each one of the single helices was individually embedded into the POPC bilayer system. Lipids which overlapped with the helix were removed and finally, the patch resulted in 122 lipids (6344 atoms). After hydrating the system with 3655 water molecules (10965 atoms), it underwent steps of minimization (5000 steps of steepest decent and 5000 steps of conjugated gradient) and equilibration for a total of 7.9 ns. Equilibration was achieved by gradually increasing the temperature from 100 K to 200 K and after that, to 310 K, whilst keeping the peptide fully restrained with k = 1000 kJ mol^-1^ nm^-2^. The first two simulations (100 K and 200 K) were run for 200 ps, the last simulation (310 K) was run for 1.5 ns. Holding the system at 310 K, the restraints, imposed by a force constant k on the peptide, were released in 4 steps (k = 500 kJ mol^-1^ nm^-2^, k = 250 kJ mol^-1^ nm^-2^, k = 100 kJ mol^-1^ nm^-2^, and k = 25 kJ mol^-1^ nm^-2^), running each of the steps for 1.5 ns. The unconstrained systems were submitted to production runs of 50 ns. The p7 monomer was embedded in a patch of 276 lipids (14352 atoms) and hydrated with 8746 water molecules (26238 atoms). As soon as the loop was included, two additional chloride ions were added to compensate charges resulting from the residues (Lys-33 and Arg-35) within the loop. The simulated boxes consist of 276 lipids and 8744 water molecules.

The root mean square fluctuation (RMSF) of Cα atoms was calculated from data derived from the last 20 ns of the 50 ns-simulations. The tilt and kink values were measured over the center of mass from the Cα atoms of residues 5–8, 11–14 and 17–21, as well as 1–4, 12–15 and 29–32 for TMD_1-32_ (here residue number according to the sequence used in the simulation software) and also averaged over the frames of the last 20 ns of the simulation. The kink angle is the angle set by the two ends of the helices. Any kink would result in an angle lower than 180°.

### Assembly of the monomers

The starting structure of TMDs for assembly was the average structure over the backbone atoms of the 50 ns MD simulations. Rotational and translational motions were removed by fitting the peptide structure of each time frame to the starting structure. The program g_covar from the GROMACS-3.3.1 and 4.0.5 packages was used for the calculations (Krüger & Fischer 2009).

The derived helices were assembled using a protocol reported earlier (Krüger & Fischer 2009; Hsu & Fischer 2011). The two helical backbone structures were aligned symmetrically towards a central axis. To sample the whole conformational space of the bundles, each of the degrees of freedom were varied stepwise: (i) inter helical distance in steps of 0.25 Å covering 9 to 15 Å; (ii) rotational angles around the helical axis in steps of 5° covering 360°; (iii) tilt in steps of 2° covering −36 to +36°. The side chains were linked to the backbone, for each position. The side chain conformation was chosen to be the most likely one for a given backbone position and referenced in the MOE library. A short minimization (15 steps of steepest decent) followed the linking (Chen et al. 2011). In this way, 2985984 conformers of the p7 M_NL_ were generated and stored in a data base for further analysis. The potential energy of each conformer was evaluated, according to the united-atom AMBER94 force field. The structure with the lowest energy is taken for further analysis. To mimic the bilayer environment, the dielectric constant was set to 2.

The simulations were run on a DELL i7-930 workstation and a 28 core Opteron based computer cluster with Infiniband interconnects.

Plots and pictures were made with VMD-1.8.7 and MOE-2008.10 and 2010.10.

### Docking approach

FlexX 2.0 (http://www.biosolveit.com) was used to dock small molecule ligands to the proteins. Flexible ring conformations were computed by CORINA, a 3D structure generator interfaced with FlexX. Two atoms, from each protein, were selected to define the center of a sphere with a radius of 20 Å. All atoms of the proteins were situated within the spheres. The drugs, BIT225 (N-(5-(1-methyl-1H-pyrazol-4-yl) naphthalene-2-carbonyl) guanidine), amantadine (1-adamantylamine) and rimantadine (1-(1-adamantyl) ethanamine) were obtained from the PubChem compound library (pubchem.ncbi.nlm.nih.gov). *N*N-DNJ (N-nonyl-deoxynojirimycin) was generated and minimized with the MMFF94x using the MOE building software. The scoring of the FlexX module is based on a geometry-based scoring (Böhm 1994), calculating estimated free energies (Rarey et al. 1996). The HYDE module of LeadIT 2.1.2 (http://www.biosolveit.com) was used to derive a rescoring based on the Gibbs-Helmholtz equations describing hydration and desolvation of the individual atoms in the ligand-protein complex (Schneider et al. 2011). The energies values for the two terms, hydration and desolvation, were calculated in respect to hydrogen bonding, hydrophobic interactions and desolvation energies, as well as further calibrated using octanol/water partitioning data. The protocol also includes two optimization procedures, which optimize the hydrogen bond network between the ligand-protein complex and a numerical optimization algorithm.

## Results

### MD simulations of individual wild type and mutant TMDs

**Figure 1:**
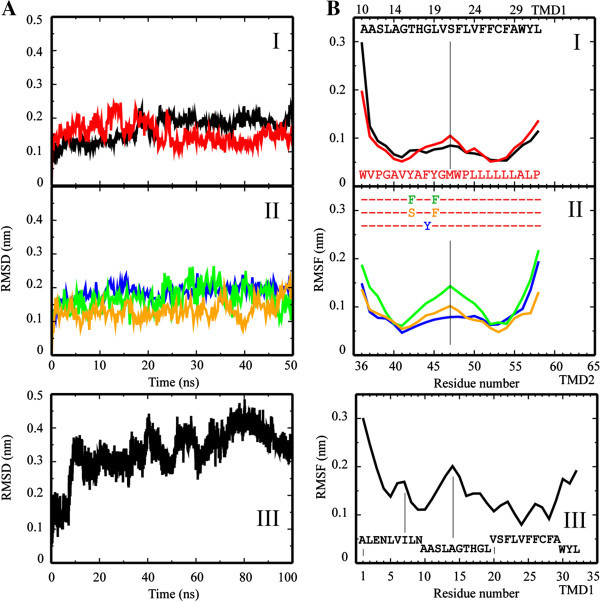
**Root mean square deviation (RMSD) and fluctuation (RMSF) data of the single TMDs.** RMSD **(A)** and RMSF plots (**B** I, II, III) of the Cα atoms of the single TMDs embedded in a fully hydrated lipid bilayer. Values for TMD1_10-32_ and TMD2_36-58_ are shown in black and red, respectively (**A**I); values for the mutants are shown in blue (TMD2_36-58_F44Y), green (TMD2_36-58_Y42F/Y45F) and orange (TMD2_36-58_Y42S/Y45S) (**A**II), those for TMD1_1-32_ are shown in (**A**III). The respective RMSF values are shown in the same color scheme as mentioned. Residue numbers according to the sequence number in the protein (see Materials and Methods).

The TMDs of p7 (see also Patargias et al. (2006)) are generated as ideal helices, individually embedded into a fully hydrated lipid bilayer and run for 50 ns (TMD1_10-32_ and TMD2_36-58_) and 100 ns (TMD1_1-32_). The root mean square deviation (RMSD) values of the Cα atoms of all TMDs investigated, level off after a short rise within the first few nanoseconds (Figure 1A). The RMSF calculations reveal a *w*-like pattern for all TMDs (Figure 1B, I – III). At the N-termini of wild type TMD1 and TMD2, RMSF values are higher than at the C-termini (Figure 1B, I). In TMD1, Ser-21 and Phe-22 exhibit maximal RMSF values. Large fluctuations are found for a Gly-46/Met-47/Trp-48 motif of TMD2. Residues within the head group region and at the interface of the hydrophobic core of the membrane hardly fluctuate. RMSF values for TMD1_1-32_ identify a maximum fluctuation for residue Ala-14 and smaller fluctuations for residues Val-6 and Ile-7 (Figure 1B, III).

A stretch of mutant TMD2-Y42/45F from residue Phe-44 to Leu-50, including the GMW motif, adopts values above 0.1 nm (Figure 1B, II, green). On both sides of the center peak, lowest values remain at similar values like the ones found for WT TMD2. RMSF values for TMD2-Y42/45S follow the pattern of TMD2 (Figure 1B, II, orange), whilst TMD2-F44Y shows a more extended stretch of fluctuating residues, almost similar to TMD1_10-32_ (Figure 1B, II, blue).

The *w*-shape of the RMSF curve reflects the mobility of the lipid bilayer in its central core. Replacing hydrophilic residues by others (TM2-Y42/45S) or increasing the hydrophilic stretch by another residue (TM2-F44Y), does not alter the dynamics of the residues. A lengthening of the hydrophobic stretch in the center of the TMD (TM2-Y42/45F) goes parallel with increased dynamics of the residues within the hydrophobic core of the membrane.

DSSP analysis (Dictionary of Secondary Structure of Proteins) reveals that the GMW motif of TMD2 adopts a turn like structure (Additional file 1: Figure S1A). The analysis of TMD1_1-32_ indicates two types of kinetics: (i) a stepwise development of turn motifs emerging from Ala-14 *via* His-17/Gly-18 towards Ser-21/Phe-22/Leu-23 and (ii) from Ala-14 in a single step towards Val-6/Ile-7 (Additional file 1: Figure S1B).

Averaged kink for TMD1_10-32_ (156.2 ± 9.4)° is lower than for TMD2_36-58_ (142.6 ± 7.3)° (Table 1), but the tilt (14.1 ± 5.5)° is higher than for TMD2_36-58_ (8.9 ± 4.2)°. Lengthening the hydrophobic core of TMD2 as in TMD2-Y42/45F results in a large kink of the helix (153.0 ± 11.3)° but lower tilt towards the membrane normal ((7.8 ± 3.9)°). Increasing hydrophilicity within TMD2 (TMD2-F44Y) results in very large kink (136.1 ± 21.0)° and tilt angles (20.8 ± 4.9)°. Whilst decreasing the size of already existing hydrophilic residues within TMD2 (TMD2-Y42/45S) rather affects the kink (162.0 ± 8.1)° than the tilt (8.5 ± 3.5)° angle, when compared with TMD2_36-58_. The large kink of TMD1_1-32_, (147.5 ± 9.1)°, is due to the conformational changes towards its N terminal side. The averaged tilt angle adopts a value of (20.1 ± 4.2)° and with this it is, on average, larger than the tilt of TMD1_10-32_.

**Table 1:** **Averaged kink and tilt angles in degree of the individual TMDs and the TMDs of the monomer**

	Kink [°]	Tilt [°]
**Individual TMDs**		
TMD1_10-32_	156.2 ± 9.4	14.1 ± 5.5
TMD2_36-58_	142.6 ± 7.3	8.9 ± 4.2
TMD1_1-32_	147.5 ± 9.1	20.1 ± 4.2
TMD2-F44Y	136.1 ± 21.0	20.8 ± 4.9
TMD2-Y42/45S	162.0 ± 8.1	8.5 ± 3.5
TMD2-Y42/45F	153.0 ± 11.3	7.8 ± 3.9
**Monomer**		
**M**_**NL**_		
TMD1	161.7 ± 5.6	24.4 ± 6.9
TMD2	143.1 ± 8.4	28.8 ± 11.8
**M**_**L**_		
TMD1	159.2 ± 4.3	12.8 ± 4.3
TMD2	159.6 ± 5.7	18.6 ± 2.9

The values represent averages taken over the entire length of each of the simulations.

**Figure 2:**
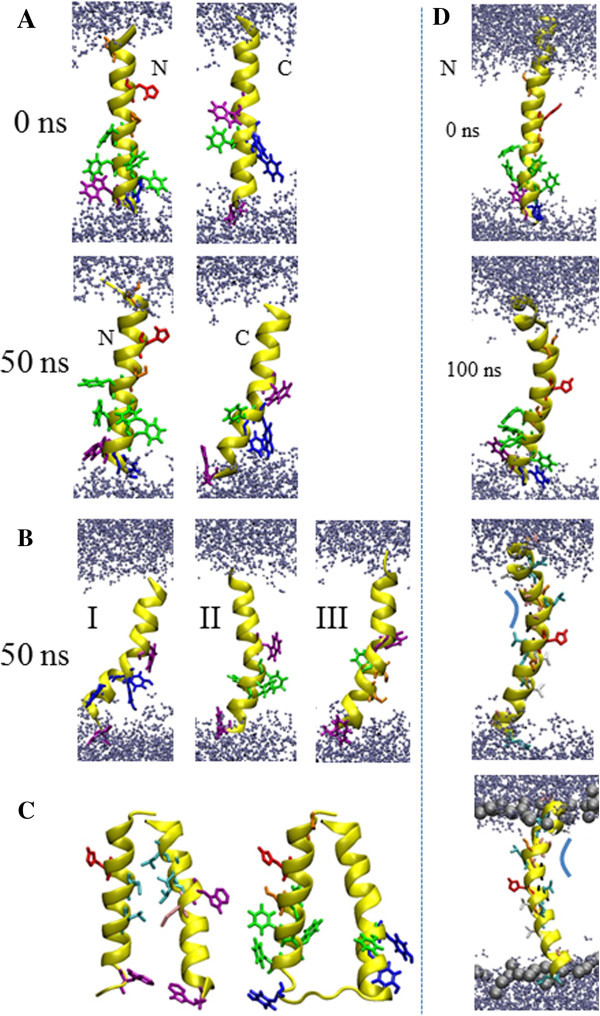
**Graphical representation of the TMDs.** Snapshots of TMD1_10-32_ (**A**, left column) and TMD2_36-58_ (**A**, right column) are shown at 0 ns and 50 ns. The individual mutant TMDs (left), (middle), (right) are presented with structures at 50 ns **(B)**. The lowest energy structures of the assembled monomers (assembled with MOE) without (left) and with loop (right) are outlined **(C)**. The left monomer highlights the leusines (light blue). The backbone is shown in yellow for all structures. TMD1_1-32_ is shown at 0 ns and 100 ns, as well as in different perspectives and with some residues indicated **(D)**. Histidine (red), phenylalanines (green), tyrosines (dark blue), tryptophans (magenta), methionine (pink), valines (white), glycines (black), leusines (light blue) and serines (orange) are marked in stick modus. Water molecules are drawn in blue, using a ball-stick modus. Lipids are omitted for clarity. The bar in **(D)** indicates the backbone exposed side of the helix to the membrane.

Visible inspection of the simulation data reveals that TMD1_10-32_ remains straight in the lipid bilayer and TMD2 kinks and tilts away from the membrane normal in a 50 ns simulation (Figure 2A, left and right). Water molecules are found in close proximity to the hydroxyl group of Y-42/45 for TMD2 (Figure 2B, I). Mutating an additional tyrosine into the N terminal side of TMD2 (TM2-F44Y) results in an increased interaction of the tyrosines with the phospholipid head group region and leads to penetration of water molecules into this region. These dynamics are not observed for TMD2-Y42/45S and TMD2-Y42/45F (Figure 2B, II and III). TMD1_1-32_ adopts a strong bend structure with a complex kink/bend motif starting from Ala-14 towards the N terminal side (Figure 2D). The motif is driven by integration of the N terminal side into the phospholipid head group region. During the 100 ns simulation, a ‘groove’ develops, in which the backbone is exposed to the environment due to accumulation of alanines and a glycine at one side of the helix (Figure 2D, lower two panels, highlighted with a bend bar).

### Assembly of the p7 monomer (TMD1_10-32_ and TMD2_36-58_) and MD simulations

Assembling TMD1 and TMD2 reveals a monomer, M_NL_, with the lowest energy at 452.5 kcal/mol, a minimum distance of 11.6 Å, a tilt of −8° and a narrow energy valley for the rotational angles of both TMDs (Figure 2C and Additional file 2: Figure S2). The monomer assembles enabling Leu-19 (10) and Leu-23(14) of TMD1, as well as Leu-50, -52 and -53 of TMD2, to intercalate, forming a hydrophobic pocket (Figure 2C, left). Tryptophans at both ends of the helices (Trp-30 (TMD1) and Trp-36 (TMD2)) cause the two helices to stay apart giving the overall assembly a conical shape (Figure 2C, left and right). The widening towards the linking region is also supported by the bulky valines of TMD2, Val-37 and −41.

In 150 ns MD simulations of the monomer, either without the linking loop or in the presence of it, show RMSD values of around 0.25 nm. During the course of the simulation, the RMSD of the monomer without loop also reaches values of around 0.3 nm.

**Figure 3:**
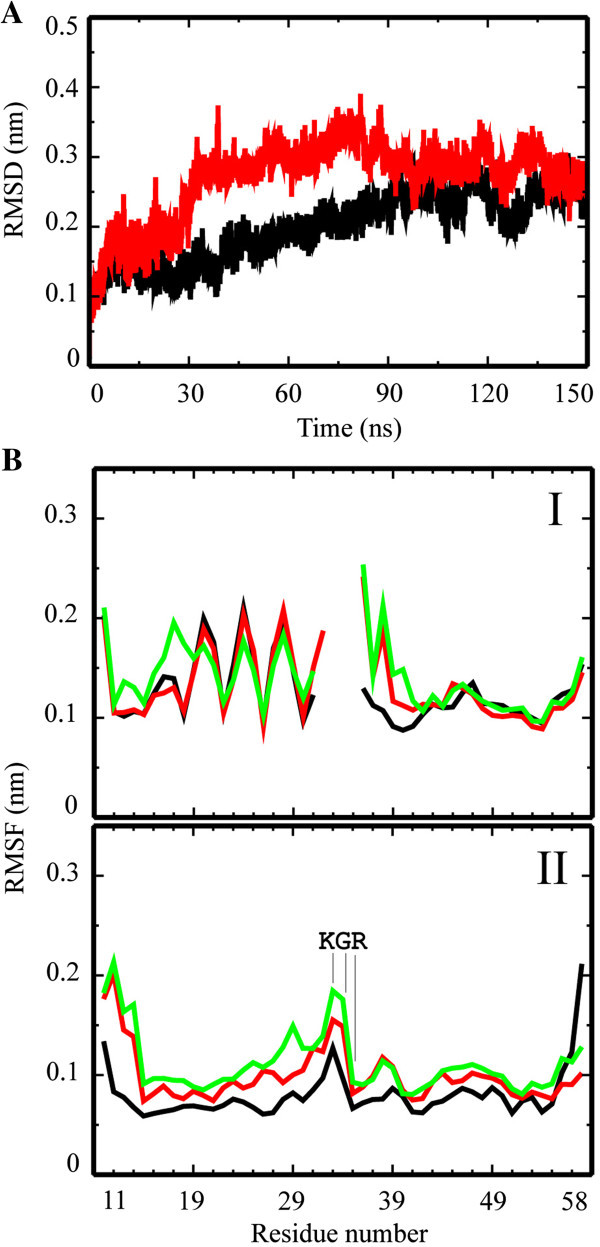
**Root mean square deviation (RMSD) and fluctuation (RMSF) data of the monomers.** RMSD plots of the simulations of the monomers without (red) and with (black) loop **(A)**. The respective time resolved RMSF data of the simulations without (I) and with (II) loop are shown for frames at 50 ns (black), 100 ns (red) and 150 ns (green) **(B)**. Residue numbers according to the sequence number in the protein (see Materials and Methods).

The RMSF values for TMD1 in M_NL_ ‘oscillate’ between 0.2 and 0.1 nm, especially on the C terminal side (Figure 3, I). The ‘amplitude’ decreases over the course of the simulation. This pattern does not affect the helicity of the TMD (Additional file 3: Figure S3, I). For TMD2, high RMSF values (around and above 0.2 nm) are calculated for the first five residues on the N terminal side. The values level around 0.1 nm towards the C-terminal side. For M_L_, all RMSF values level around 0.1 except for the first 5 residues on the N-terminus and the last two residues on the C-terminus (Figure 3, II). Throughout the simulation, the fluctuation of the residues at the C-terminal side of TMD1 increases, reaching almost 0.2 nm for Lys-33 and Gly-34. The value for Arg-35 is calculated to be about 0.1 nm. Similar to M_NL_, TMD2 develops a *w*-like pattern of its RMSF values, identifying a dynamic hydrophobic core region.

**Figure 4:**
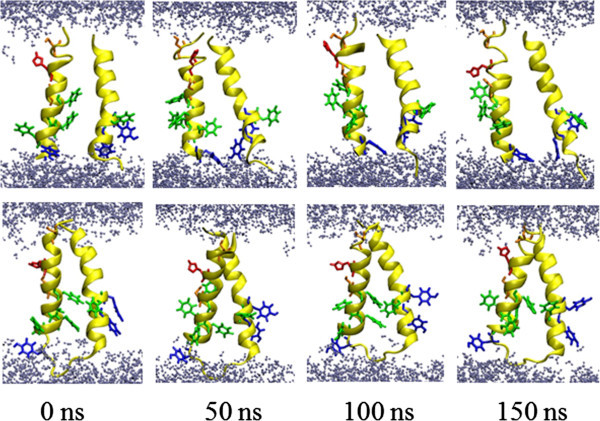
**Graphical representation of the monomers.** Snapshots of the 150 ns simulations of the monomers without (top row) and with loop (botom row) separately embedded into hydrated lipid bilayers. The backbone is shown in yellow. Histidine (red), phenylalanines (green), tyrosines (dark blue), serine (orange) are shown in stick modus. Water molecules are drawn in blue using a ball-stick modus. Lipids are omitted for clarity.

Following the trajectories of the MD simulations, the two TMDs of M_NL_ adopt a slightly higher tilted structure (24.4° and 28.8° for TMD1 and TMD2, respectively) than the TMDs in M_L_ (12.8° and 18.6° for TMD1 and TMD2, respectively; Figure 4 and Table 1). In M_NL_, kink angles of the TMDs adopt values of 161.7° for TMD1 and 143.1°, for TMD2 they are almost the same (around 159°) for M_L_. Consequently, the loop induces conformational constraints, resulting in a moderate and almost similar tilt of both TMDs. At the current stage of the simulation of the monomer, the tyrosines of TMD2 move into the hydrophobic core region of the lipid bilayer and attract water molecules towards the end of the simulation (Figure 4, lower panel).

### Docking approach with the p7 monomer

**Figure 5:**
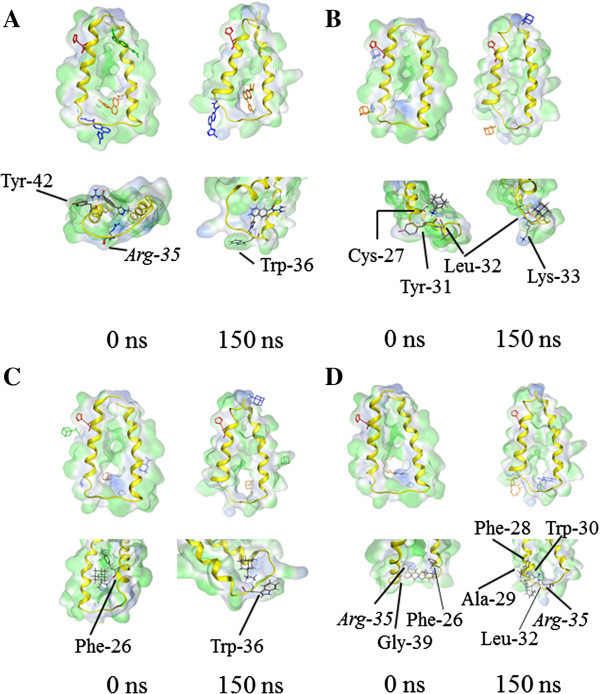
**Small molecule drug docking to the monomers.** Docking of small molecule drugs to the monomer with loop taken from 150 ns MD simulation: BIT225 **(A)**, amantadine **(B)**, rimantadine **(C)** and *N*N-DNJ **(D)**. For each drug the best pose is shown in orange, the second best pose in blue and the third best pose in green.

Docking the small molecule drug BIT225 to M_NL_, taken from the MD simulation at 0 ns, shows the first binding site (−16.7 kJ/mol, see Table 2) to be located towards the side of the loop (data not shown). A second site is found at the C terminal side of TMD1 (−13.7 kJ/mol) and a third site at the C terminal side of TMD2 (−12.6 kJ/mol). For the structure at 150 ns, the top three sites are changed so that the first site is at the N terminal side (−17.7 kJ/mol), the second at the C terminal side of TMD1 (−16.2 kJ/mol), and the third site (−13.9 kJ/mol) at the N terminal side of TMD2. Interactions of the sites are driven by hydrogen bonding of the guanidinium group with the amide bond of the protein backbone. Refined calculations using HYDE, leaves the sequence for the structure at 0 ns (see Table 2): for the 150 ns structures though, the best pose becomes the third in rank ((values in kJ/mol): -17.7/-14.4 kJ/mol (FlexX (Score^F^)/HYDE (Score^H^)) (Table 2). For M_L_, the best pose remains faced towards the loop for both structures (the one at 0 and the one at 150 ns) and the second site remains faced towards the C-terminal side of TMD1 (Figure 5A). A third site at the C-terminus of TMD2, found for the structure taken from 0 ns, is not identified after 150 ns. The best poses with M_NL_ show that the pyrazol group establishes hydrogen bonds with the side chain of Arg-35 and the backbone nitrogen of Trp-36. The binding affinities, including refined calculations, are as low as approximately −20 kJ/mol for the best sites at the 0 ns (−21.6/-16.5 kJ/mol) and 150 ns structures (−23.8/-27.0 kJ/mol). Refined calculations do not replace the best poses.

**Table 2:** **Calculated poses** (**FlexX 2**.**0**) **and estimated binding affinity calculated with HYDE module** (**FlexX**) **for the drugs BIT225**, **amantadine**, **rimantadine and*N*N**-**DNJ**

Compound	Time	No loop		Loop		TMD1_1-32_	
		Score^F^	Score^H^	Score^F^	Score^H^	Score^F^	Score^H^
		[kJ/mol]	[kJ/mol]	[kJ/mol]	[kJ/mol]	[kJ/mol]	[kJ/mol]
BIT225	0	−16.7	−21.7	−21.6	−16.5		
		−13.7	−15.9	−16.8	1.4		
		−12.6	−6.7	−13.9	−9.8		
	150	−17.7	−14.4	−23.8	−27.0	−14.3	−8.5
		−16.2	−16.4	−20.2	−14.4	−12.1	−13.4
		−13.9	−15.9			−11.1	−8.6
ssAmatadine	0	1.4	−2.7	−5.0	−9.3		
				−0.5	−10.8		
	150	−2.3	−8.5	−3.7	−5.7	−2.0	−9.9
		−1.6	0.6	−3.0	−3.8	−0.5	−8.0
Rimantadine	0	−3.7	−2.4	−4.8	−7.9		
		−3.1	−10.0	−4.2	−2.4		
		−1.6	−8.1	−2.6	−7.7		
	150	−2.8	−15.6	−4.7	−10.1	−3.8	−15.9
		−1.9	−7.0	−4.6	−7.2	−1.2	−12.6
		−1.6	−15.0	−3.8	−12.4	−0.9	−7.4
*N*N-DNJ	0	−2.0	−8.2	−7.8	−16.1		
		−1.1	−21.9				
	150	−0.9	−8.0	−7.1	−8.9	2.5	−21.3
		−0.3	−39.3	−4.1	−14.6	2.9	−20.7
						4.8	−11.2

The energies of the best poses of each cluster are shown for the respective structures at 0 ns and 150 ns (Time). All values are given in kJ/mol. ‘Score^F^’ refers to the values from FlexX 2.0, ‘score^H^’ to those from HYDE.

The sites of amantadine at different structures of M_NL_ are identified to be with the N-terminus of TMD2 for the best pose of the structure at 0 ns, but found at the N (TMD1)/C-terminal sides (TMD2) in the structure at 150 ns, forming hydrogen bonds with the backbone (data not shown). In the presence of the loop (M_L_), amantadine also poses at the site of the loop (Figure 5B). With M_L_, amantadine forms hydrogen bonds with the backbone carbonyls of residues from TMD1 (Cys-27, Tyr-31, Leu-32 (structure at 0 ns) and Leu-32, Lys-33 (structure at 150 ns). The best pose of binding of rimantadine with M_NL_ is identified to be *via* its amino group, with the backbone carbonyl of either Trp-48 (0 ns structure) or the hydroxyl group of the side chain of Ser-12 (150 ns structure) (data not shown). The best pose for rimantadine in M_L_ is with the backbone of Phe-26, which is within the TMD (structure at 0 ns) and the backbone of Trp-36, which is within the loop of the structure at 150 ns (Figure 5C). The second best pose with the 150 ns structure is found to be towards the C-terminal side of TMD2. In all cases, the binding affinities for amantadine and rimantadine are in the range of −10 kJ/mol to 0 kJ/mol (Table 2). For amantadine docked to M_NL_, the order reverses position 2 and 3 for rimantadine (0 and 150 ns structure). For amantadine docked to M_L_, the order reverses for the structure at 0 ns. At this second site (first in respect to HYDE), the interaction is driven by hydrogen bonding of the amino group of amantadine with the backbone carbonyls of His-17 and the hydroxyl group in the side chain of Ser-12 (data not shown). For the M_L_ structure at 150 ns with rimantadine, the third pose becomes the best one when recalculating the energies with HYDE. In this pose, hydrogen binding of the amino group of rimantadine with the carbonyl backbone of Tyr-33 together with hydrophobic interactions between adamantan and the aromatic rings of Tyr-42 and −45 (data not shown) is found.

Docking of *N*N-DNJ onto M_NL_ identifies the best pose between the two ends of the TMDs towards the side of the loop (data not shown). Backbone carbonyls of Tyr-42, Ala-43 and Gly-46 form hydrogen bonds *via* the hydroxyl groups of the iminosugar moiety with the structure at 0 ns. The hydrogen bonding of Tyr-42 serves as an acceptor for two off the hydroxyl groups of the ligand. The carbonyl backbone of His-17, as well as the backbone NH groups of Gly-15 and Leu-19 both serve as hydrogen acceptors and donors, respectively, in TMD1 at 150 ns. Based on the refined calculation of the binding affinities, the best poses based on FlexX of −2.0/-8.2 kJ/mol (0 ns structure) and −0.9/-8.0 kJ/mol (150 ns structure)) become the second best for both structures, when recalculating with HYDE (−1.1/-21.9 kJ/mol (0 ns) and −0.3/-39.3 kJ/mol (150 ns)). The large values of −21.9 and −39.3 kJ/mol are due to the large number of hydrogen bonds (each hydroxyl group forms a hydrogen bond with carbonyl backbones and side chains in combinations with favorable hydrophobic interactions (data not shown). The best pose of *N*N-DNJ with M_L_ is in the loop region *via* hydrogen bonds of the hydroxyl group with carbonyl backbone group of Phe-26 and Gly-39 in the 0 ns structure (Figure 5D). In addition, one hydroxyl group of *N*N-DNJ forms a hydrogen bond with the side chain of Arg-35. The binding affinities are calculated to be −7.8/-16.1 kJ/mol. In the 150 ns M_L_ structure, a maximum of hydrogen bond partners are suggested: carbonyl backbone groups of Phe-28, Ala-29, Trp-30 and Leu-32, as well as side chain of Arg-35 for the best pose (−7.1/-8.9 kJ/mol). In addition to that, the aliphatic chain is surrounded by hydrophobic side chains of Ala-29 and Tyr-31. Refined calculations put the second pose into the first rank (−4.1/-14.6 kJ/mol). Similarly, in this pose, hydrogen bonds are formed with the backbone carbonyls of Gly-34 and Try-36. The aliphatic tail is embedded into a hydrophobic pocket of Leu-32, Lys-33, Gly-34 and Trp-36 (data not shown). *N*N-DNJ is the only ligand which interacts with carbonyl backbones of the residues of TMD1_1-32_ (150 ns structure) closer to the N terminal side: Ala-10, -11 and Gly-15. The alkyl chain adopts van der Waals interactions with small residues such as Ala-14, Gly-15/18.

All small molecules mentioned, show best binding sites with TMD1_1-32_ towards the C-terminal side and at its end: no pose at the extended N-terminal side is identified at this stage. Both types of calculations of the binding affinities leave all best poses in the same order (Table 2).

Docking indicates that the C-terminal side and the loop region impose a high potential drug binding site. Considering M_L_ and all binding affinities for ranking the compounds, the following sequence can be suggested: BIT225 < *N*N-DNJ < Amantadine ≈ Rimantadine.

## Discussion

### Bio-inspired pathway translated into feasible computational steps

Membrane proteins are manufactured at the site of the endoplasmic membrane *via* interplay between ribosome and translocon. The protein is released into the membrane through a side passage of the translocon. The stoichiometry of the overall reaction is: one ribosome per translocon generates one protein. Consequently, the proteins generated along this pathway are the monomers which have to oligomerize within the lipid membrane in order to generate a functional ion channel. It is assumed, that between manufacturing the monomer and theassembly into an oligomer, there is ‘enough time’ to ‘equilibrate’ the monomer in accordance with the respective environmental conditions. In case of p7, the protein needs to be cleaved from the polyprotein precursor. Finally, the respective monomer have to assemble with other p7 monomers to form a pore. With this in mind, the modeling strategy is chosen to (i) generate the individual helices of p7 and relax the structures briefly *via* MD simulations in a fully hydrated lipid bilayer, (ii) assemble the resulting two helices into a monomer using a docking approach, which mimics the lipid environment, and (iii) relax the monomer further *via* MD simulations.

The effect of selected structures on a docking approach is evaluated through choosing monomer structures at 0 ns and 100 ns.

### Simulations of TMD1 with two different lengths

The role of the individual helical segments within TMD1 can be evaluated by simulating the domain with two different lengths. TMD1_10-32_ is chosen based on a consensus derived from several secondary structure prediction programs (SSPPs). The longer helix TMD1_1-32_ includes the N-terminal part which also has been predicted by only one of the SSPPs, e.g. SPLIT4 (Patargias et al. 2006), but is now identified by NMR studies (Cook & Opella 2011; Montserret et al. 2010). There is consensus among the two simulations in as much as the weakly fluctuating Ser-21/Phe-22 of the shorter TMD1_10-32_ is mobile in simulations of TMD1_1-32_. Due to the extended helix which remains in the motif during 100 ns MD simulations, the most flexible part is moved one helical turn further towards the N terminal side, spiking around Ala-14. This leaves the residues towards the C-terminal side from Ala-14 onwards gradually declining in their mobility. Consequently, the resulting assembled structures with the shorter TMD1 and TMD2 are a reliable motif for the monomer and the respective bundles.

This reasonable choice of the shorter TMDs is supported further by the feature, that the backbone of TMD1_1-32_ is exposed to the environment due to the accumulating alanines (Ala-10/-11/-14) and glycines (Gly-15) on one side of the helix. The assembled models of TMD1_10-32_ with TMD2 show, that TMD2 ‘uses’ this exposed part to approach the backbone of TMD1 closely to form the tepee-like structure.

According to the RMSF data, the ‘naked’ section of TMD1_1-32_ allows some flexibility within this region, making it susceptible to entropic or enthalpy driven effects. Therefore, it is possible that this region is an important section for gating related conformational changes.

Analysis of the DSSP plot of TMD1_1-32_ reveals stepwise conformational changes which almost ‘jump’ over one helical turn to the next leaving the original one back in a helical conformation. These ‘jumps’ seem to follow n+1 and n+2 helical turns and imply a ‘self-healing’ of the helix.

### Simulations with mutants and their impact on the structure

Due to the tyrosines 42 and 45, TMD2 experiences a considerable kink combined with a moderate tilt. The kink angle is increased when mutating the hydrophobic residue Phe-44 into tyrosine. The increase of the kink occurs due to the ‘snorkeling’ of the tyrosines for the hydrophilic head group region and the aqueous phase. The snorkeling effect (usually used in context with lysines (Strandberg & Killian 2003)), is accompanied by a further insertion of the rest of the part of the helix which is directed towards the other leaflet into the hydrophobic part of the membrane. Removing the hydroxy groups, as in TM2-Y42/45F, reduces the snorkeling and with it the kink and tilt. Smaller hydrophilic residues, such as serines, do not have a big impact on either the kink or the tilt angle of the helix. Serine rather forms hydrogen bonds with the backbone to compensate unfavorable interactions with the hydrophobic environment of the lipid membrane, than to interact with the lipid head groups and water molecules (after a while). It is concluded, that hydrophilic residues, accumulated on one side of a TM helix, lead to attract water molecules to compensate for hydrogen bonding and charges, and a tearing further into the hydrophobic core region of its other side. The consequence is a considerable kink or bend of the helix. In the monomer, the bending of TMD2 is preserved, when running the monomer with a linker.

If further bending is hampered, the hydrophilic residues could alternatively force water molecules into the lipid bilayer. Other studies show, that water is being dragged into the membrane when a helix containing arginine residues is positioned in the membrane (Dorairaj & Allen 2007). More generally, a hydrophilic helix, fully inserted in the lipid membrane, completely hydrates itself during a 100 ns MD simulation (Hong et al. 2012).

### Comparison of the structural model with data from NMR spectroscopy

Two monomeric structures (Cook & Opella 2011; Montserret et al. 2010) and a bundle structure (OuYang et al. 2013) have been reported which are derived from NMR spectroscopic experiments.

Solid state NMR spectroscopic analysis of p7 (genotype J4, 1b) expressed as a fusion construct in *Escherichia coli*, purified and reconstituted into DHPC (1,2-diheptanoyl-sn-glycero-3-phosphocholine) let four helical segments to be suggested within the lipid bilayer (Cook & Opella 2011). The four segments can be distinguished by their mobility. NMR data allow the statement, that segments from residue 5 – 15 (TMD1) and from 41 – 48 (TMD2) are more mobile than segments 17 – 27 (TMD1) and 49 – 57 (TMD2). The latter two segments adopt measurable tilts of 25° and 10°, respectively. In the present study, TMD1_10-32_ includes residues 10 – 15 of the reported experimental study. The *w*-shaped RMSF plots, calculated from MD simulations of the individual helices, support the experimental results of 4 segments separated by higher fluctuating residues (Figure 1B, I). The ‘separating’ residues are around Val-20/Ser-21/Phe-22 for TMD1_10-32_, Ala-14 for TMD1_1-32_ and the GMW-motif for TMD2. Whilst the residues in TMD1_10-32_ are shifted by one helical turn towards the C-terminus compared to the NMR data, the residues in TMD1_1-32_ and TMD2_36-58_ almost match the residues found in the NMR experiments. The separating residues in TMD2 are conserved in the simulations of the monomer with and without loop (Figure 3B). Simulations of the monomer with loop reveals fluctuations around Pro-38 within the N-terminal part of TMD2. This is similar to high fluctuations found in the simulations of M_NL_ indicating higher mobility of this segment in accordance with the NMR findings. According to the MD simulations, TMD2 seems to adopt a larger tilt than TMD1 with a stronger kink of TMD2 than TMD1.

In another study, a computational model based on combined CD and NMR spectra is proposed (Montserret et al. 2010). Synthesized peptides, corresponding to full length protein and the individual TMDs of p7 protein (sequence based on HCV-J of genotype 1b), have been reconstituted into detergents such as 100 mM dodecylphosphocholine (DPC) or sodium dodecyl sulphate (SDS), or dissolved in TFE/H_2_O mixtures. The structural models of the individual TMDs have been subject of protein-protein docking using GRAMM program (Tovchigrechko & Vakser 2006) to generate a monomer. The assembled TMDs have consequently been linked by four amino acids modeling a hairpin motive using VMD software. The spectra support the motif of two helical segments towards the N-terminal side and a shorter helix at the C-terminal side followed by a mobile segment. Both of the sections are separated by a hairpin like motif. The computational model of the monomer has been run for 40 ns MD simulation (NAMD, CHARMM27) in a fully hydrated lipid bilayer. From the MD simulations in this study, a flexible region of Gly-15 to Gly-18 is suggested, which matches with higher RMSF values, which were calculated for the MD simulations of the monomer in the present study (Figure 3BI,II). In this study, the flexible region is positioned within one helix turn (Leu-13 to Gly-15) towards the N terminal side (Figure 1B, IV). The staging motif of alternating aromatic residues from TMD1 and TMD2 to define the TMD1-loop-TMD2 region (Montserret et al. 2010) has not been found in the present study.

Taken together, the described computational model of monomeric p7 matches the experimental data in several aspects. Thus, the working protocol described in this study, is a worthwhile tool to assess structural features of membrane proteins. Simulation on TMD1_1-32_ fits into the experimental findings. Consequently, a region of residues with short side chains N-terminal from His-17 is flexible and exposes the backbone, potentially for gating kinetics.

Comparison of TMD2-NMR with TMD2_36-58_ reveals that the NMR structure is comparable within the error margins in respect to kink and tilt angles. The similarity holds especially the C terminal side, despite the additional residues on either side of TMD2-NMR as well as their unwinding. This unwinding obscures the identification of the w-shape of the RMSF values, since the fluctuation of the additional five helices result in high values.

### Binding site in the loop region

The sensitivity of p7 towards inhibitors has been reported to be strain specific (StGelais et al. 2009; Griffin et al. 2008). Bilayer recording data report on a blockage of p7 by *N*N-DNJ which is more effective than blockage by amantadine and rimantadine (Steinmann et al. 2007b). Also, strain specific tests in cell culture reveal activity of these compounds (Griffin et al. 2008). Resistant mutations, observed upon adminstration of the two typs of drugs affect residues (i) Leu-20 (into L20F) induced by adamantanes and (ii) Phe-25 (into F25A) induced by iminosugars (Foster et al. 2011). These sites are within TMD1. Application of a docking approach using Autodock, on a heptameric bundle and a monomer, support a potential binding site within the TM region of p7. The poly leusine motif (Leu-50 to Leu-55) has been identified to be sensitive to amantadine (Cook & Opella 2010). In the present docking study, the site for amantadine interaction with p7 does not match these experimental findings (Cook & Opella 2010; StGelais et al. 2009; Griffin et al. 2008).

In a previous computational docking approach of the hexameric p7 bundle, a binding site for amantadine *via* hydrogen bonding with the carbonyl group of Ser-21 has been proposed (Patargias et al. 2006). With the binding residues presented in this study, amantadine is very close to the binding of Ser-21, as reported earlier. The discrepancy may rather occur due to the use of the monomer in this study, than the bundle as in the afore mentioned study (Patargias et al. 2006).

The prime site of interaction for all small molecule drugs investigated, including BIT225, in this study, is the loop region by forming hydrogen bonds with carbonyl backbones. In case of the iminosugars, this site in the loop region is possibly less favorable than for BIT225, even though a number of hydrogen bonds can be formed. The disfavor may be because of the aliphatic chain of *N*N-DNJ, which has to cope with the unfavorable position. The chain could interact with hydrophobic pockets in the protein, though this comes with some entropic costs. For amantadine and rimantadines, the same situation may hold with some minor advantages in as much as the hydrophobic part of these molecules may not get many restrictions in conformational flexibility upon binding. In contrast to e.g. *N*N-DNJ, amantadine and rimantadine can form fewer numbers of hydrogen bonds, what then compensates the entropic costs arising for *N*N-DNJ upon binding. BIT225 seems as the most favorable molecule, in respect of entropic costs. Experiments with mutants in this region would be necessary to proof the proposed mechanism of binding. What do the results mean for a potential drug? The potent drug should interact with sensitive amino acids, preferentially with its backbone, in the loop region.

What are the biological consequences of the interaction with the water exposed sites of the protein? It has been shown, that residues in the loop region, Lys-33 and Arg-35, are important for the functioning of the protein (Steinmann et al. 2007b). Binding of any drug *via* interacting with the backbone of the protein would hamper the dynamics of the loop and with it, possibly, the free energy involved in the mechanism of function. In the specific case of BIT225, the interaction is indeed identified to be with the backbone of the highly important residues Arg-35. This may explain the successful antiviral activity of this compound compared to amantadine and one of its derivatives.

## Conclusions

Computational structural modeling of biological molecules, for which experimentally derived date is rare, is a challenging task. Two ‘key stones’ are at hand when starting the endeavor, (i) the membrane protein to be discussed is inserted into the lipid membrane *via* the translocon complex, and (ii) the two stage folding model of membrane proteins, which suggests, that the secondary structure is generated prior to any further assembly process. According to the present study, the side chain residues are further responsible for the ‘fine tuning’ of the secondary structure. The tyrosines of TMD2 in p7 are important residues defining the shape of the helix and with it the structure of the monomer.

With the loop region, p7 exposes itself to the aqueous environment making this part of the protein an ideal target site. The investigated small molecule drugs in this study indicates, that the interaction could be *via* hydrogen bonding with main chain atoms of sensitive amino acids in the loop.

## Electronic supplementary material

Additional file 1: Figure S1: DSSP plots of the individual TMDs embedded into hydrated lipid bilayers reporting a 50 ns MD simulation: TMD1_10-32_ (I), TMD2_36-58_ (II), TMD2_36-58_F44Y (III), TMD2_36-58_Y42F/Y45F (IV), TMD2_36-58_Y42S/Y45S (V) and TMD1_1-32_ (VI). The colors encode for α-helix (blue), 3_10_-helix (grey), turn (yellow), bend (green), and coiled structure (white). Residue numbers according to the sequence number in the protein (see Materials and Methods). (JPEG 945 KB)

Additional file 2: Figure S2: Energy plots of the assembly of the monomer. Energies are plotted over distance (top left), tile (top right), and the rotational angles of the two TMDs (bottom left and right). (TIFF 3 MB)

Additional file 3: Figure S3: DSSP plots of the monomer without (I) and with (II) loop embedded into hydrated lipid bilayers. The residues numbers are counting the residues number (see Materials and Methods). The colors encode for α-helix (blue), 5-helix (pink), 3_10_-helix (grey), β-sheet (red) and β-bridge (black), turn (yellow), bend (green), and coiled structure (white). (TIFF 4 MB)
