# Supplementary figures and images for: Computational modeling of the p7 monomer from HCV and its interaction with small molecule drugs
Source: Springerplus. 2013 Jul 18;2:324. doi: 10.1186/2193-1801-2-324 (PMC3724979; doi:10.1186/2193-1801-2-324)

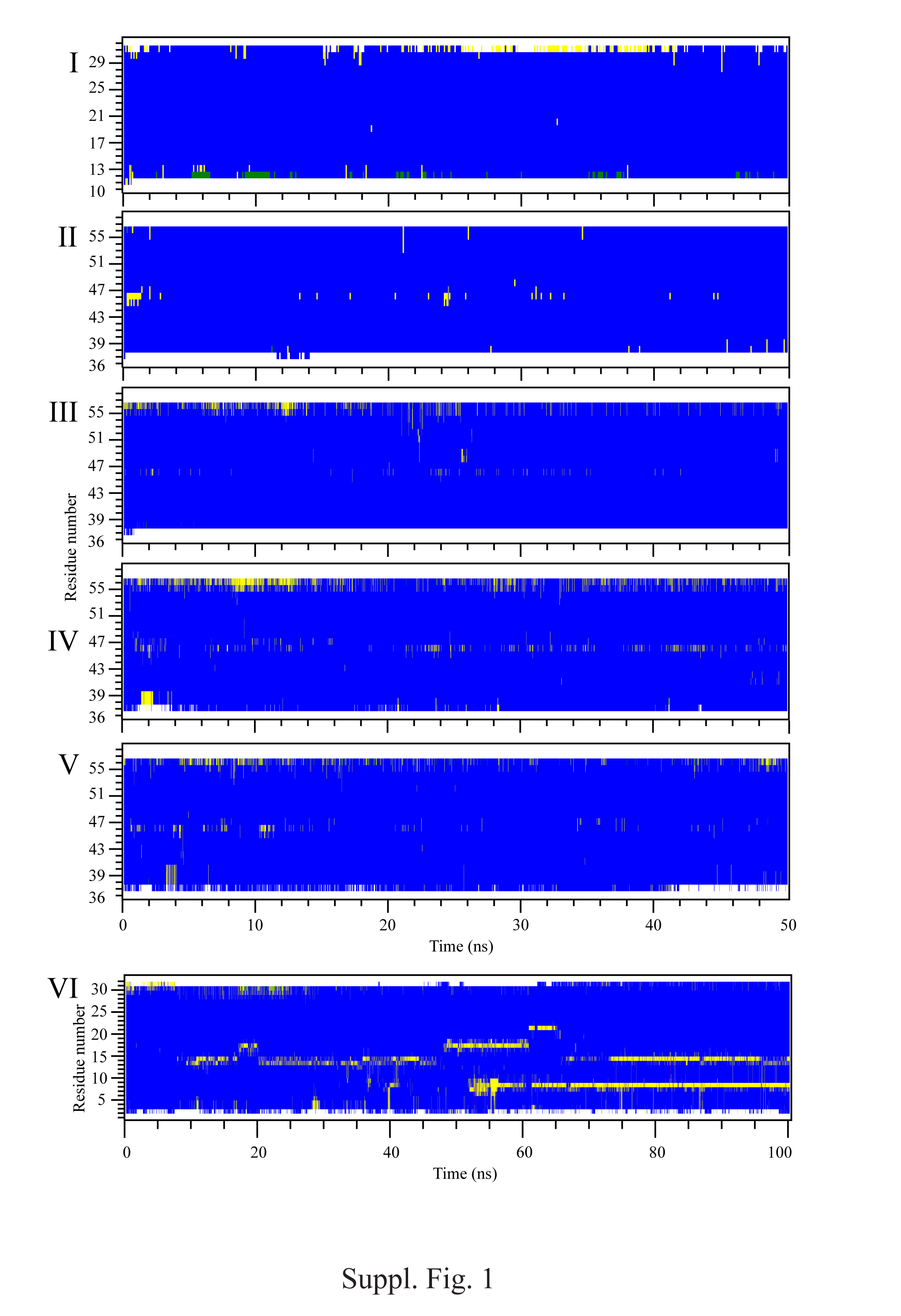

Supplement: Supplementary file 1 — Additional file 1: Figure S1: DSSP plots of the individual TMDs embedded into hydrated lipid bilayers reporting a 50 ns MD simulation: TMD110-32 (I), TMD236-58 (II), TMD236-58F44Y (III), TMD236-58Y42F/Y45F (IV), TMD236-58Y42S/Y45S (V) and TMD11-32 (VI). The colors encode for α-helix (blue), 310-helix (grey), turn (yellow), bend (green), and coiled structure (white). Residue numbers according to the sequence number in the protein (see Materials and Methods). (JPEG 945 KB) [file 40064_2013_397_MOESM1_ESM.jpeg]

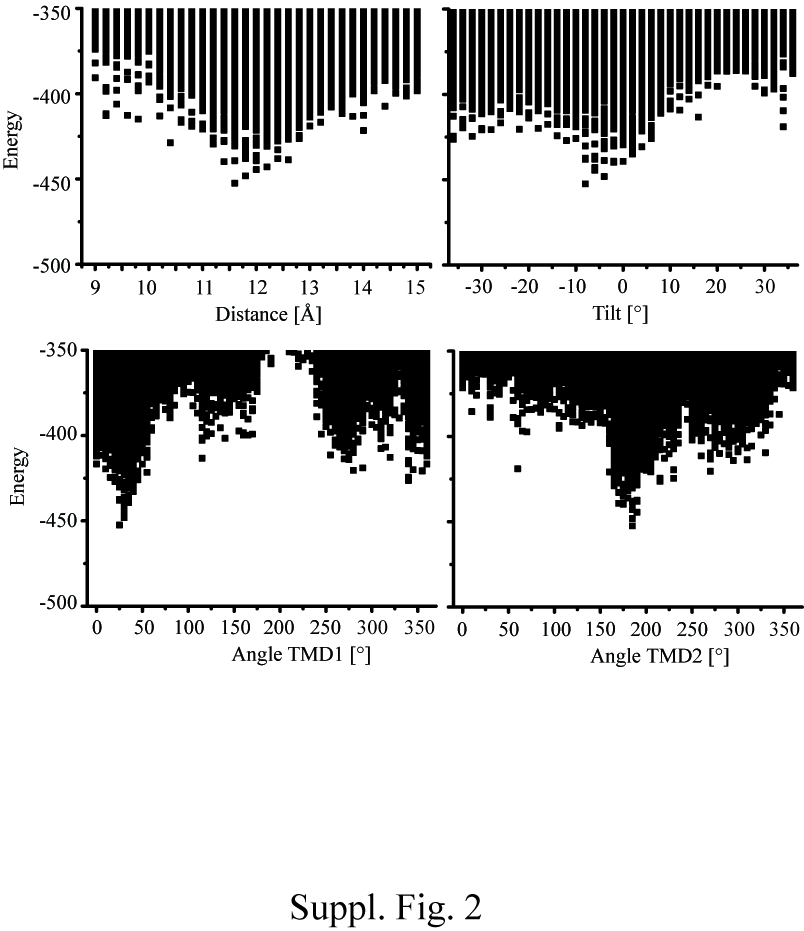

Supplement: Supplementary file 2 — Additional file 2: Figure S2: Energy plots of the assembly of the monomer. Energies are plotted over distance (top left), tile (top right), and the rotational angles of the two TMDs (bottom left and right). (TIFF 3 MB) [file 40064_2013_397_MOESM2_ESM.tiff]

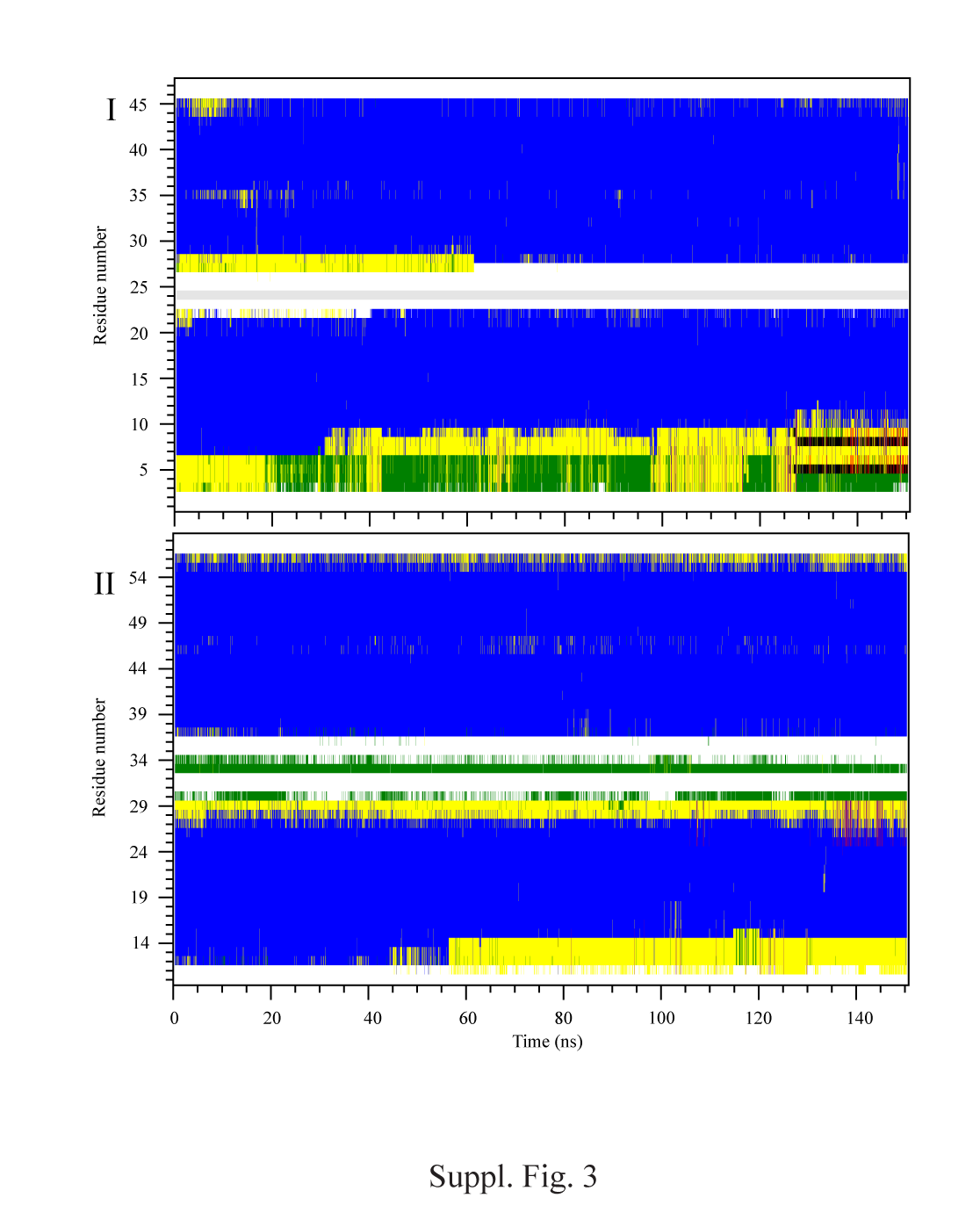

Supplement: Supplementary file 3 — Additional file 3: Figure S3: DSSP plots of the monomer without (I) and with (II) loop embedded into hydrated lipid bilayers. The residues numbers are counting the residues number (see Materials and Methods). The colors encode for α-helix (blue), 5-helix (pink), 310-helix (grey), β-sheet (red) and β-bridge (black), turn (yellow), bend (green), and coiled structure (white). (TIFF 4 MB) [file 40064_2013_397_MOESM3_ESM.tiff]
